# Disseminated Tuberculosis Masquerading as Alcoholic Liver Disease

**DOI:** 10.7759/cureus.58902

**Published:** 2024-04-24

**Authors:** Anjana Ledwani, Ulhas Jadhav, Pankaj Wagh, Ashwin Karnan

**Affiliations:** 1 Respiratory Medicine, Jawaharlal Nehru Medical College, Datta Meghe Institute of Higher Education and Research, Wardha, IND

**Keywords:** adenosine deaminase, ascites, pancytopenia, portal hypertension, tuberculosis

## Abstract

Tuberculosis (TB), caused by the bacteria *Mycobacterium tuberculosis*, is a highly infectious and prevalent disease. It is the leading cause of death among communicable diseases and the fifth leading cause of all diseases in India. The diagnosis can be challenging due to the disease’s unique appearance and various presentations. Disseminated TB is characterized by the involvement of two or more non-contiguous sites resulting from hematogenous extension of the disease. Clinical confirmation of the diagnosis of disseminated TB is based on bacteriological or histological evidence. Based on various studies, there is evidence that satisfactory results are obtained from treatment with first-line anti-tubercular drugs. When there is a delay in diagnosis and treatment, it can become a life-threatening condition. We present a case of a 38-year-old alcoholic male who presented with generalized edema, pain, and distension of the abdomen. According to the initial presentation, the provisional diagnosis made was alcoholic liver disease, but it was later diagnosed as disseminated TB with sputum-positive pulmonary TB with abdominal involvement in the form of ascites and hepatosplenomegaly along with hematological involvement as pancytopenia. The patient started showing drastic improvement after the initiation of anti-tubercular therapy.

## Introduction

The presence of two or more noncontiguous sites or isolation of *Mycobacterium tuberculosis* from blood or bone marrow as a result of the hematogenous dispersion of *M. tuberculosis*, which can arise from a progressive primary infection or the reactivation of a latent focus with subsequent spread, is known as disseminated tuberculosis (TB) [1]. Adult cases of disseminated TB have been identified more frequently recently. This is because immunosuppressive treatments are being given for a range of illnesses, and the increased prevalence of immune suppression brought on by acquired immunodeficiency syndrome (AIDS) is to blame. Other conditions like alcohol use disorder (AUD), chronic liver disease, chronic kidney disease, diabetes mellitus, and malnutrition diminish the body’s immune response and hence lead to the dissemination of infection. An unusual clinical appearance frequently causes a delay in diagnosis. Hence, a high degree of suspicion is necessary for the accurate diagnosis and timely management of the disease, as any delay can be detrimental.

Miliary TB, a type of disseminated TB, is represented by very small tubercles that are visible on gross pathology and have an appearance and size similar to millet seeds. It is caused by a vast lymphohematogenous dissemination of *M. tuberculosis* bacilli.

## Case presentation

A 38-year-old male presented to the emergency department with chief complaints of fever with chills, generalized edema, pain, and distension of the abdomen for the past two months. The patient also had complaints of breathlessness for two months, which was insidious in onset, and had gradually progressed from grades I-II to grade III of Modified Medical Research Council (mMRC) over the past 15 days. He was a chronic alcoholic and had taken his last drink 15 days back. The patient had no other significant past or personal history. On admission, his physical examination revealed an overall cachexic appearance with a body mass index (BMI) of 16.3 kg/m^2^, pallor present, pulse rate of 120 beats/minute, blood pressure of 100/70 mmHg, respiratory rate of 26 breaths/minute, and oxygen saturation on room air at 86%, requiring oxygen support of 4 L/minute. On examination of his respiratory system, bilateral coarse crepitations were present.

**Figure 1:**
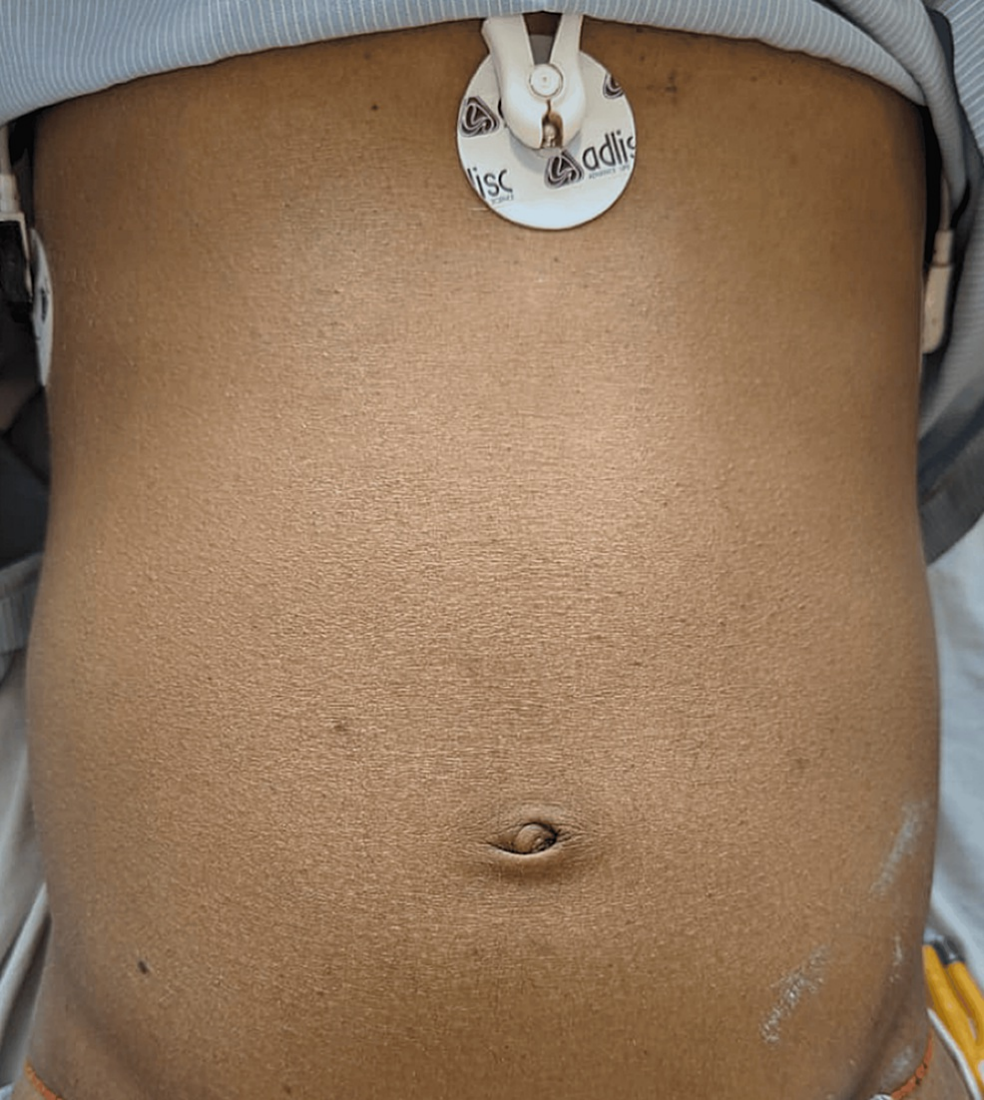
Clinical image of the patient showing abdominal distension suggestive of ascites

On per abdominal examination, the patient had splenomegaly and gross ascites with the presence of fluid thrill (Figure 1). 

Relevant blood investigations were done, which are depicted in Table 1 [2].

**Table 1:** Blood investigations of the patient MCH, mean corpuscular hemoglobin; MCV, mean corpuscular volume; ESR, erythrocyte sedimentation rate; AST, aspartate aminotransferase; ALT, alanine aminotransferase; GGT, gamma glutamyl transferase

Investigations	Values	Reference range
Hemoglobin	9.6 gm%	13-17 gm%
Total leucocyte count	3500/cu.mm	4000-10000/cu.mm
Platelet count	0.42 lacs/cu.mm	1.50-4.0 lacs/cu.mm
MCV	62.4 fL	80-100 fL
MCH	20.2 Pico-gm	27-32 Pico-gm
ESR	120 mm/1 h	1-10 mm/1 h
Urea	24 mg/dL	20-40 mg/dL
Creatinine	0.8 mg/dL	0.8-1.3 mg/dL
Sodium	128 mmol/L	135-145 mmol/L
Potassium	3.7mmol/L	3.5-5.0 mmol/L
Total bilirubin	1.4 mg/dL	0.2-1.3 mg/dL
AST	51U/L	5-30 U/L
ALT	42 U/L	5-30 U/L
Albumin	2.4 g/dL	3.5-5.0 g/dL
Globulin	3.2 g/dL	2.0-3.5 g/dL
GGT	32 U/L	6-50 U/L

Viral markers for hepatitis B and C were negative.

**Figure 2:**
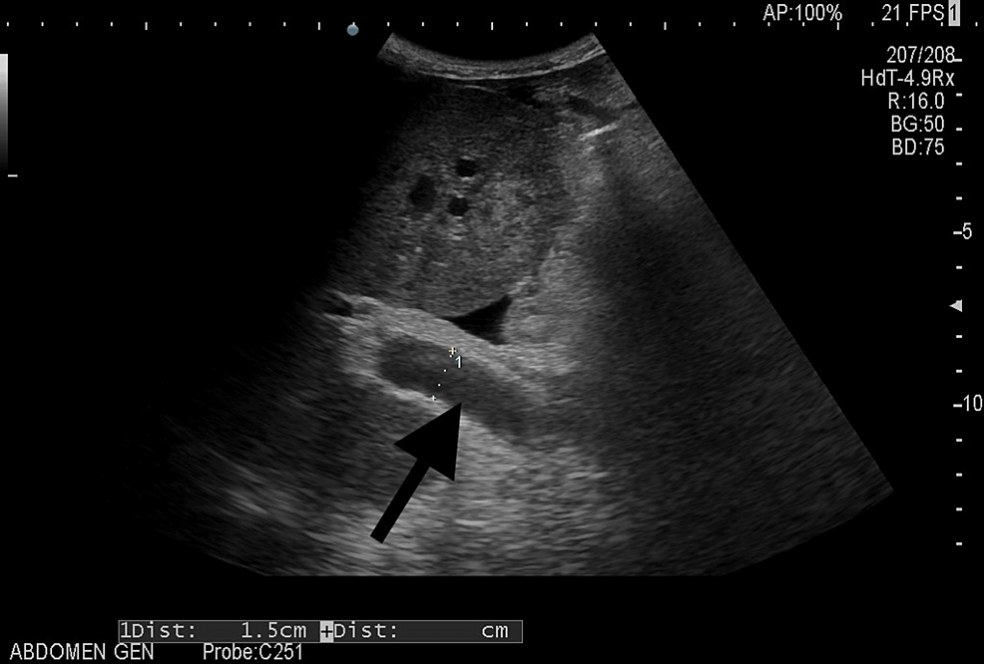
Ultrasonography of the abdomen showing a dilated portal vein measuring 1.5 cm, suggestive of portal hypertension

Ultrasonography of the abdomen showed normal echotexture of the liver with gross ascites, splenomegaly, and tortuous splenic vein with dilated portal vein with perisplenic collaterals (Figure 2).

**Figure 3:**
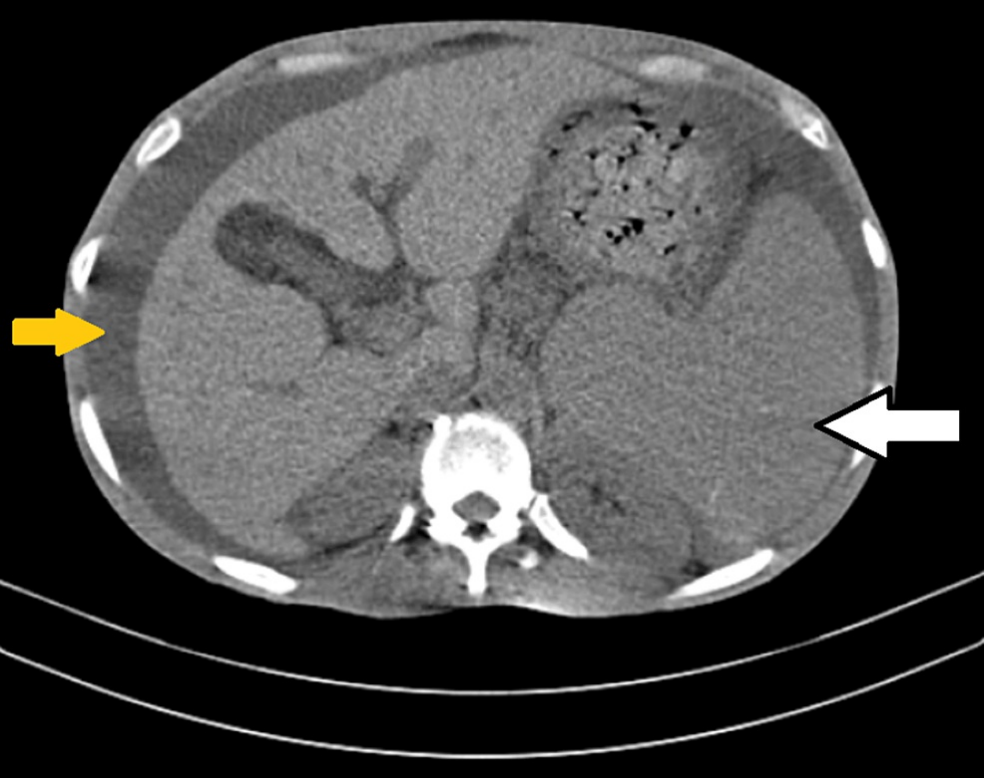
Computed tomography of the abdomen with a white arrow showing splenomegaly and a yellow arrow showing ascites

CT abdomen image of the patient is shown in Figure 3.

Ascitic fluid tapping was done under aseptic precautions and sent for investigations, which are shown in Table 2.

**Table 2:** Ascitic fluid investigations ADA, adenosine deaminase; TLC, total leucocyte count; LDH, lactate dehydrogenase; SAAG, serum albumin ascites gradient; CBNAAT, cartridge-based nucleic acid amplification test; AFB, acid-fast bacilli

Investigations	Values
ADA	47 U/L
TLC	200 cells/cu.mm
Polymorphs	30%
Lymphocytes	70%
LDH	67 IU/L
Glucose	52 mg%
Protein	2.0 gm%
pH	7.5
SAAG	0.4 gm%
CBNAAT	Positive
AFB culture	Positive

The serum albumin ascites gradient (SAAG) of the patient was 0.4 gm% (less than 1.1 gm%), which rules out portal hypertension due to chronic liver disease. Ascitic fluid adenosine deaminase (ADA) was high and positive on the cartridge-based nucleic acid amplification test (CBNAAT), suggestive of abdominal TB. Rifampicin resistance was not detected.

**Figure 4:**
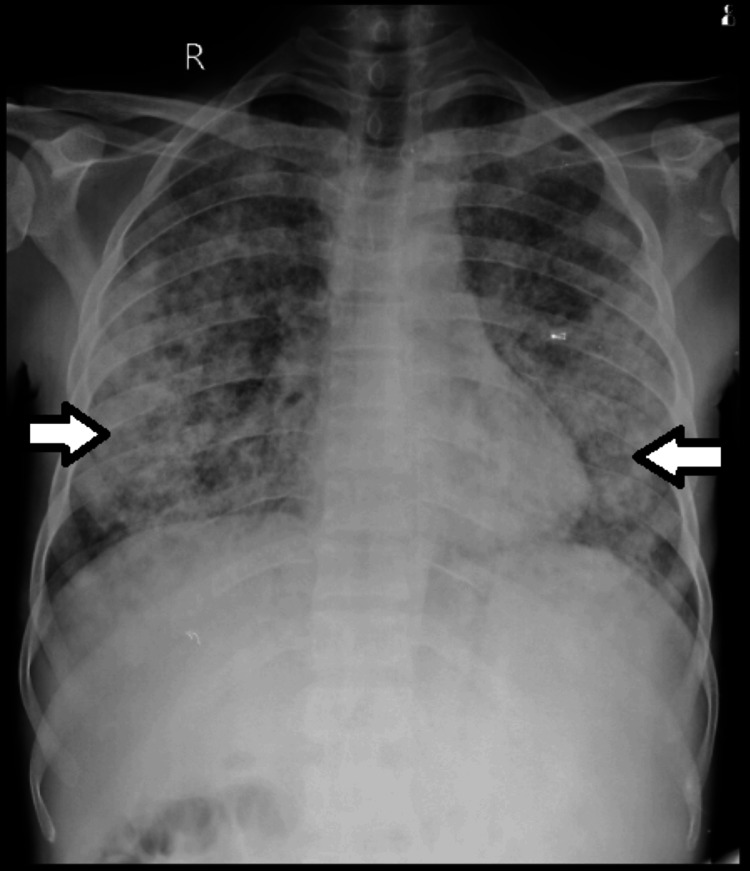
Chest X-ray of the patient showing bilateral diffuse non-homogenous nodular opacities

The chest X-ray of the patient is shown in Figure 4.

**Figure 5:**
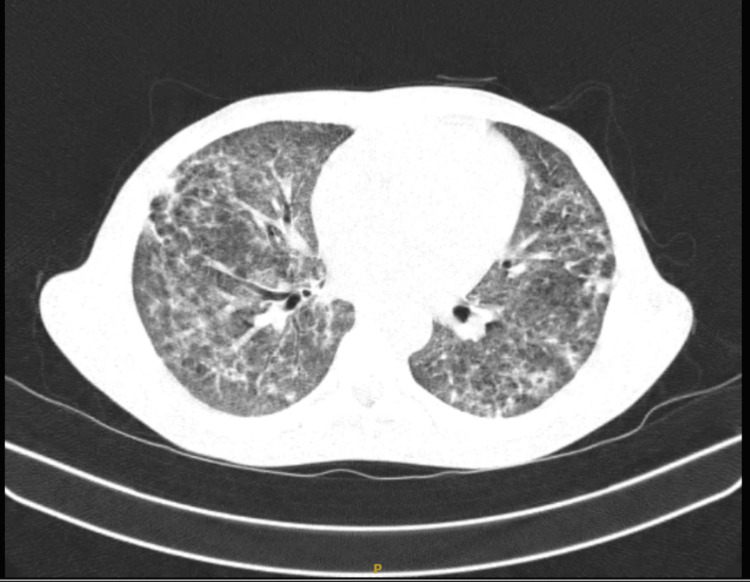
HRCT scan of thorax showing bilateral reticulonodular shadows with tree-in-bud appearance in the bilateral lower lobe HRCT, high-resolution computed tomography

High-resolution computed tomography (HRCT) scan of the thorax of the patient is shown in Figure 5.

**Figure 6:**
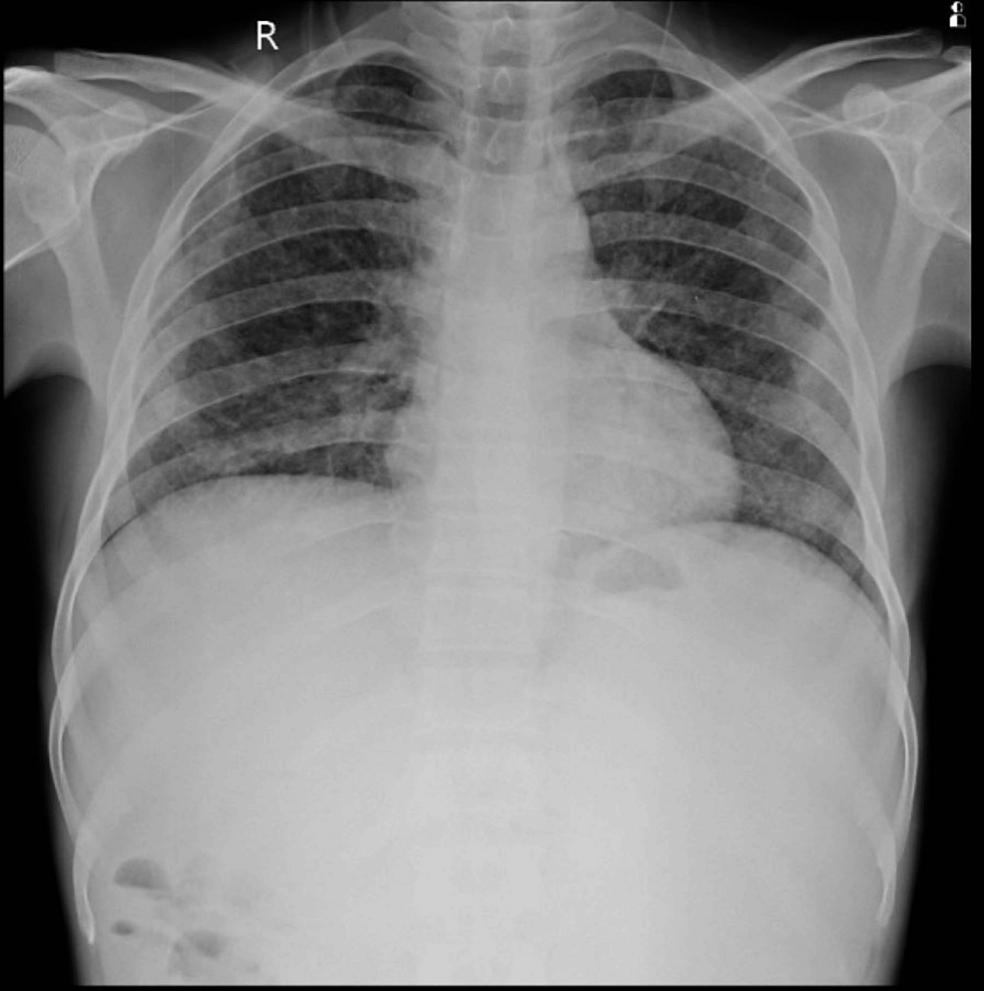
Chest X-ray of the patient showing radiological improvement

A sputum examination for acid-fast bacilli was done, which was positive, and in Truenat (nucleic acid amplification test), *M. tuberculosis* was detected with no resistance to rifampicin. A diagnosis of disseminated TB was made. The patient was started on anti-tubercular treatment, which comprised a fixed-dose combination of tab isoniazid (75 mg), tab rifampicin (150 mg), tab pyrazinamide (400 mg), and tab ethambutol (275 mg). The patient was advised to take three tablets of the fixed-dose combination once/day according to the weight band (35-49 kg). The patient was reviewed after three weeks, and repeat ultrasonography of the abdomen showed resolution of ascites and clinical improvement after initiation of treatment. At the two-month follow-up, the chest X-ray of the patient showed complete resolution of opacities (Figure 6). 

## Discussion

TB is the second most common infectious killer globally, despite improvements in diagnosis and treatment. Globally, the incidence of TB in 2022 was 133 per 100,000 population per year. A total of 7.5 million new cases were diagnosed with TB in 2022, which is the highest number since WHO began monitoring [3]. It is caused by M. tuberculosis, an acid-fast bacilli described by Robert Koch in 1882 [4]. Ziehl-Neelsen and Auramine stains are used to identify the bacilli. It is characterized by the formation of caseating granulomas consisting of Langhans giant cells in tissues.

Disseminated TB is the term used to describe the concurrent involvement in at least two non-contiguous organ locations of the body or the involvement of the bone marrow or blood [5]. Although the precise incidence of disseminated TB worldwide remains unknown, it is estimated to represent less than 2% of all TB cases and approximately 20% of extrapulmonary TB cases [6]. Miliary TB is a type of disseminated TB that arises from a large-scale hematogenous spread of tubercle bacilli. This causes small, discrete foci that are more or less equally distributed in the lungs and other viscera, typically the size of millet seeds (1-2 mm) [7]. Patients with disseminated TB present with varied symptoms and signs and often pose as difficult to diagnose the condition.

Numerous complications can arise from pulmonary TB. Hemoptysis is caused by bleeding from the pulmonary, intercostal, and bronchial arteries. Spontaneous pneumothorax may result from a rupture of the lung cavity or a subpleural focus. Inflammation of lymph nodes may result in bronchial tree compression and bronchiectasis. Severe untreated pulmonary TB can cause gangrene, necrosis, and significant lung volume loss. It has also been noted that lung cancer risk is elevated by TB. Septic shock and persistent pulmonary aspergillosis are less frequent consequences [8]. Complications of disseminated TB include polyserositis, hyponatremia due to SIADH, acute respiratory distress syndrome, sepsis with septic shock, and adrenocortical insufficiency. 

The first line of defense between hepatocytes and blood is made up of endothelial cells of the hepatic ducts. The endothelial cells of the liver identify and intercept the activated T lymphocytes carrying exogenous antigens by generating distinct cell adhesion molecules. Additionally, the liver produces the proteins involved in both innate and adaptive immune responses. All of these functions are impaired in patients with chronic liver disease, which increases the risk of infection. As TB is an endemic disease in India, clinicians need to have a low threshold for diagnosis, especially in immunocompromised patients [9]. For more than 200 years, lung infections and the consequences they cause have been related to AUD. It has been demonstrated that AUD is a separate risk factor for TB and that the risk of TB increases linearly with alcohol use. The immune function of the alveolar macrophage, which is the lung’s innate immune effector and the first line of defense against Mtb, is compromised by alcohol abuse. Additionally, long-term alcohol consumption increases oxidative stress in the alveolar region, which could stimulate Mtb growth [10]. 

Differential diagnoses for my patient include congestive cardiac failure, viral hepatitis, malignancy, alcoholic liver disease, nephrotic syndrome, TB, and hepatic vein outflow obstruction. Seromarkers for hepatitis were negative. The echocardiography of the patient was normal. Liver enzymes and gamma glutamyl transferase (GGT) were normal; hence, alcoholic liver disease was ruled out. There was no evidence of proteinuria. Ultrasonography of the abdomen showed portal hypertension with normal echotexture of the liver, ruling out any lesion metastases to the liver. High ESR and albumin globulin ratio reversal was seen in this patient, pointing toward a chronic inflammatory state. Sputum Truenat was positive for MTB, and ascitic fluid ADA was high and positive on CBNAAT, thereby confirming the diagnosis of disseminated TB.

Wang et al. conducted a study whereby 3058 patients with TB confirmed by culture between 1995 and 2004 were found, of which 164 (5.4%) had disseminated TB. Miliary lung lesions were the most frequent radiographic finding (47.0%); after the research, 31.1% of patients had passed away. Hypoalbuminemia, hyperbilirubinemia, renal dysfunction, and postponed antituberculosis treatment were among the poor prognostic variables. Of all patients, 70.7% had clinical signs suggestive of disseminated TB [11]. 

According to a study by Vithoosan et al., when making a differential diagnosis for overblown ascites, one should take into account tuberculous peritonitis caused by disseminated TB [12]. Despite the challenge of diagnosis, diagnostic laparoscopy and biopsy are very beneficial. Early diagnosis is crucial in these situations because treatment delays may have negative consequences.

According to the abstract provided by Moatemri et al., disseminated TB was responsible for 7.6% (n = 7) of all TB cases at a military hospital’s pulmonary department (n = 92) between 2007 and 2012. The liver (28.5%), bowel (28.5%), and bone (28.5%) were the most frequently involved extra-pulmonary organs. Splenic, gastric, peritoneal, hemopoietic, cerebral, meningitis, laryngeal, and tongue were each involved in one case. Three serious complications occurred: respiratory failure and stroke in one case, medullar compression in the second, and hemophagocytic syndrome with multi-organ failure in the last [13].

## Conclusions

Disseminated TB is an important and often misdiagnosed health problem that contributes significantly to morbidity and mortality in a country, especially in an endemic country like India. This case report emphasizes that clinicians and radiologists need to have a high index of suspicion for the diagnosis of disseminated TB as the patient can present with varying and unspecific symptoms. There may be difficulty in obtaining appropriate tissue specimens, especially from inaccessible sites, and also due to the poor sensitivity of diagnostic tests available. There should not be any delay in the initiation of the treatment as it can lead to poor prognosis and high mortality.
